# Transcriptional landscape of pleural mesothelioma patients in relation to *NF2* gene mutational status

**DOI:** 10.1186/s43046-025-00284-0

**Published:** 2025-06-09

**Authors:** Carlos Orozco-Castaño, Alejandro Mejía-Garcia, Hsuan Megan Tsao, Diego A. Bonilla, Carlos Carvajal-Fierro, Ricardo Bruges-Maya, Alba Combita, Rafael Parra-Medina

**Affiliations:** 1 Instituto Nacional de Cancerología, Bogotá, Colombia; 2 McGill University, Montreal, Canada; 3 University of the Basque Country, Leioa, Spain; 4 DBSS International SAS, Bogotá, Colombia; 5 Fundación Universitaria de Ciencias de La Salud, Bogotá, Colombia

**Keywords:** Pleural mesothelioma, *NF2* mutations, Transcriptome analysis, Chemotherapeutic response, Immune infiltration, Personalized therapy

## Abstract

**Background:**

Pleural mesothelioma (PM) is an aggressive cancer with poor prognosis, often driven by asbestos exposure. Mutations in the *NF2* gene, a key regulator of the Hippo signaling pathway, are frequently observed in PM. However, their impact on tumor biology, immune infiltration, cytokine signaling, and therapeutic response remains poorly understood.

**Methods:**

Using data from The Cancer Genome Atlas, we analyzed 82 PM cases to assess the prevalence and consequences of *NF2* mutations. Logistic regression was used to evaluate associations with clinical variables, while transcriptomic differences were examined through differential expression and functional enrichment analyses. Immune and stromal infiltration were inferred via the xCell algorithm, cytokine signaling analyzed with Cytosig, and chemotherapeutic sensitivity predicted using the pRRophetic R package. Single-cell RNA sequencing data provided further insights into transcriptional patterns in *NF2*-mutated tumors.

**Results:**

*NF2* mutations were present in 22% of cases, with no significant correlations to histological subtype, stage, or age. *NF2*-mutated tumors exhibited increased infiltration of basophils, naïve B cells, and pericytes, along with altered cytokine profiles, including NRG1, TGFB3, and reduced FGF2. Differentially expressed genes, such as *MYL7* and *HOXA11*, were linked to poorer survival. Chemotherapy modeling indicated higher sensitivity to camptothecin and vinblastine in *NF2*-mutated tumors.

**Conclusions:**

*NF2* mutations influence the tumor microenvironment, transcriptional landscape, and predicted therapeutic response in PM, underscoring their potential as prognostic biomarkers. These findings support tailored therapeutic strategies targeting NF2-related pathways, including Hippo signaling and cytokine modulation.

**Supplementary Information:**

The online version contains supplementary material available at 10.1186/s43046-025-00284-0.

## Background

Pleural mesothelioma (PM) is an aggressive and often fatal cancer originating from the mesothelial cells lining the pleura. The primary etiological factor for PM is asbestos exposure, which has been well-documented to cause genetic alterations that lead to tumor development [1]. Although the pathogenesis of PM is relatively well understood, prognosis remains poor, with a median survival of less than 1 year from diagnosis [2]. This underscores the urgent need for improved diagnostic markers and therapeutic strategies. PM is characterized by a low mutational burden, though several genetic alterations in its oncogenesis have been identified and used as diagnostic markers. These include mutations in the *BAP1*, *CDKN2A*, and *NF2* genes [3, 4]. Among these, mutations in the *NF2* gene, which encodes the tumor suppressor protein Merlin, are particularly significant. The *NF2* gene plays a crucial role in regulating cell growth and proliferation through the Hippo signaling pathway [5]. Loss of *NF2* functions can lead to uncontrolled cell growth and tumor development, making *NF2* a critical factor in PM pathogenesis.

The importance of *NF2* mutations in PM extends beyond their roles in tumorigenesis. Immunohistochemical analysis of NF2 expression is an important diagnostic tool, as loss of its expression helps distinguish PM from other pleural malignancies, making it a valuable diagnostic marker [6]. Furthermore, studies have shown that *NF2* mutations can influence the tumor microenvironment by affecting immune cell infiltration and stromal interactions [7]. These findings have significant implications for both diagnostics and prognostics, as *NF2* mutations may serve as biomarkers for disease progression and therapeutic response. Transcriptome analysis offers a powerful method for investigating the differences between *NF2* mutated and wild PM tumors. By examining gene expression profiles, researchers can gain insight into the molecular pathways associated with *NF2* mutations and potentially identify therapeutic targets. Previous studies have demonstrated that *NF2* mutations are associated with distinct transcriptomic signatures, which may guide the development of personalized treatment strategies [8].

Despite recent advancements, effective therapies for PM remain limited. Current treatment options, including surgery, chemotherapy, and radiation therapy, offer modest efficacy and are associated with significant side effects [9, 10]. The identification of *NF2* mutations as a driver in PM presents significant potential for targeted therapies. For instance, drugs targeting focal adhesion kinase (FAK), which has been shown to be upregulated in NF2-deficient tumors, may offer more effective treatment options [11, 12]. In this study, we examined the relationship between *NF2* mutation status and clinicopathological features, immune and stromal cell infiltration, and chemotherapeutic response. Using publicly available genomic and transcriptomic data, combined with computational methods, we aimed to explore the role of *NF2* mutations in PM. Additionally, we aimed to characterize the transcriptional landscape of patients harboring these mutations and identify potential therapeutic targets. Our findings emphasize the need for further research and development of targeted therapies to improve outcomes for PM patients.

## Methods

### Aim

This study examines how *NF2* gene mutations affect the transcriptional landscape, immune infiltration, cytokine signaling, and chemotherapeutic response in PM.

### Data

Transcriptional RNA sequencing (RNA-seq) and clinicopathological data from MESO cohort of The Cancer Genome Atlas (TCGA) were retrieved. Clinical data were accessed through the UCSC Xena Browser (https://xena.ucsc.edu/), a comprehensive platform for exploring and analyzing TCGA datasets [13]. Single-cell RNA sequencing data were obtained from Gene Expression Omnibus (GEO) under accession number GSE190597.

### Mutational status of PM TCGA cohort

Mutational status data for the PM TCGA cohort were obtained from the cBioPortal for Cancer Genomics (http://www.cbioportal.org/) [14]. The cBioPortal provided detailed information on key mutations and their frequencies within the cohort. Specifically, the genomic positions and mutational details of the 20 patients with *NF2* mutations were retrieved for further analysis. Additionally, each variant was classified according to the American College of Medical Genetics (ACMG) guidelines [15], using the Varsome platform (https://varsome.com).

### Association of clinical variables and NF2 gene mutation status

A bivariate logistic regression was conducted to explore potential associations between the clinicopathological features of PM patients and the presence of *NF2* mutations. This statistical approach allowed for assessing the impact of variables such as histological subtype, tumor stage, age, and occupational asbestos exposure on the likelihood of *NF2* mutations. Odds ratios (OR) and *p*-values were calculated to quantify the strength and significance of these associations.

### Survival analysis

Survival analysis was performed using Kaplan–Meier plots to assess overall survival (OS) in relation to *NF2* mutational status and *NF2* expression levels. The survival module from the UCSC Xena Browser was utilized for this analysis, allowing for the visualization and statistical comparison of survival curves [16]. Kaplan–Meier plots were generated to compare survival outcomes between *NF2-*mutated and *NF2* wild-type patients.

### Differential expression analysis and functional enrichment

Differential expression analysis was performed by categorizing patients into *NF2*-mutated and *NF2* wild-type groups. The Limma Voom package [17] was employed, offering robust statistical methods for RNA-seq data analysis. A volcano plot was generated to visualize the upregulated and downregulated differentially expressed genes (DEGs), using log2 fold change and -log *p*-values to emphasize significant changes.

To investigate the biological implications of the identified DEGs, functional enrichment analysis was performed. Gene Ontology (GO) categories, including Biological Process (BP) and Molecular Function (MF), were selected for enrichment, along with the Kyoto Encyclopedia of Genes and Genomes (KEGG) pathway analysis. Additionally, transcription factors and kinase ontologies were explored to provide insights into regulatory mechanisms and potential therapeutic targets [18].

### Estimation of immune and stromal infiltration

The xCell R package was utilized to estimate immune and stromal cell infiltration within the tumor microenvironment, stratified by *NF2* mutational status. xCell applies a gene signature-based method to infer the relative abundance of various cell types, offering detailed insights into the composition of the tumor microenvironment [19].

### Prediction of chemotherapeutic response

The *pRRophetic* R package was utilized to predict chemotherapeutic responses in PM patients. This approach constructs statistical models based on gene expression and drug sensitivity data from a comprehensive panel of cancer cell lines, applying these models to gene expression profiles of primary tumor biopsies [20]. The resulting predictive models estimated drug sensitivity for various chemotherapeutic agents, enabling a comparative analysis between *NF2-*mutated and *NF2* wild-type patients.

### Prediction of cytokines

Cytokine, chemokine, and growth factor signaling activities were predicted and deconvoluted using the Cytosig platform [21]. This platform leverages a detailed database of target genes modulated by a wide range of cytokines, chemokines, and growth factors to estimate signaling activity. The Cytosig database includes a total of 64 cytokines, encompassing both chemokines and growth factors.

### Single cell analysis

We downloaded single-cell RNA-seq data from GEO under the accession number GSE190597. This dataset comprises 13 primary tumors, of which nine are epithelioid, two are biphasic, and two are sarcomatoid. Normalized and log-transformed expression matrices were analyzed using Seurat 4.4 in R. Cells with fewer than 400 genes, fewer than 1000 unique molecular identifiers (UMIs), or greater than 25% mitochondrial gene expression were filtered out. Principal component analysis (PCA) and K-nearest neighbor analysis were performed using Seurat with default parameters [22]. Cell annotations were assigned based on known markers for each cell type, as described in [23]. Uniform Manifold Approximation and Projection (UMAP) was used to visualize clusters in two-dimensional space. Next, we assessed the expression of four genes: *NF2, TENM2, TGFB3,* and *FGF2*. We conducted a subsequent analysis, including only cells classified as malignant, with the following expression pattern: no *NF2* expression and high expression of at least one of the three genes: *TENM2, TGFB3,* or *FGF2*. This was achieved using the AddModuleScore function. Clustering analysis was then performed using the FindClusters function with a resolution of 0.1. Overexpressed genes in each cluster were identified with the FindAllMarkers function [24]. We then performed the same clustering analysis for B cells. We verified these new subclusters using B-cell classical markers such as IgM, IgD, IgG, CD19, and CD38. Finally, enrichment analysis was carried out using the fgsea and msigdbr R packages [24]. In addition, we checked for NF2 intratumor heterogeneity by estimating the number of cells expressing NF2 within each tumor.

## Results

### Mutational status of NF2 gene in the PM TCGA cohort 

**Fig. 1:**
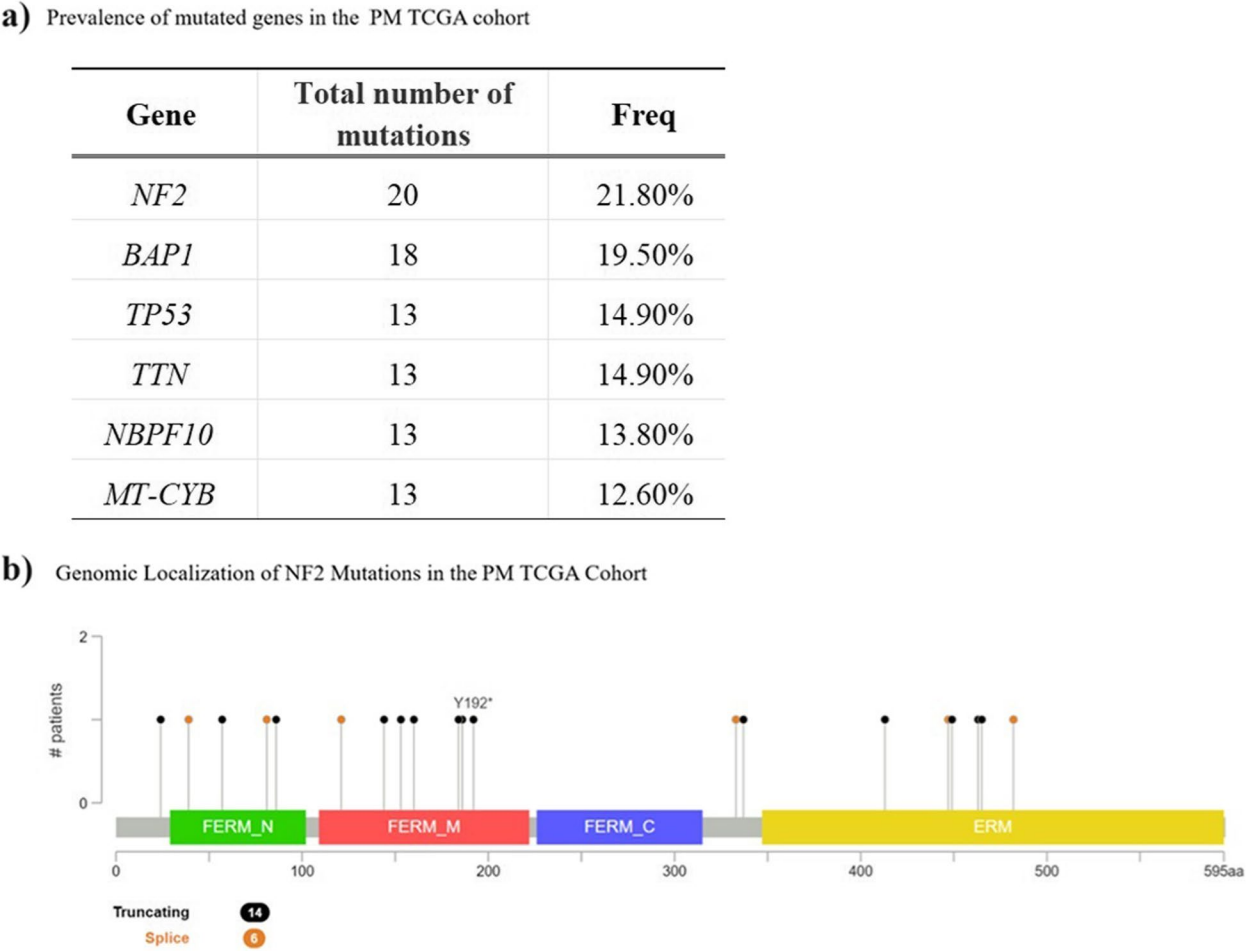
Mutational features of the PM TCGA cohort. **a** The most frequently mutated genes. **b** Diagram illustrating the genomic localization of individual patients’ mutations in the *NF2* gene. Plot b was generated using cBioportal

PM is characterized by a relatively low mutational burden, yet a subset of genes exhibits significant genetic alterations in up to 22% of patients (Fig. 1a). Among these genes, *NF2* shows the highest frequency of alterations. Despite its prevalence, the role of *NF2* in the pathogenesis of PM has not been extensively studied, although its high mutational frequency suggests a potential impact on disease progression. Analysis of the TCGA PM cohort revealed that *NF2* mutations occur at various genomic locations among patients, leading to a loss of function of the NF2 protein and impairing its tumor suppressor capabilities (Fig. 1b). This loss of function is significant, as it indicates that *NF2* mutations contribute to the malignant phenotype by disrupting key regulatory pathways involved in cell growth and survival. Notably, *NF2* mutations appear irrespective of PM histological subtype, tumor stage, or patient age (Table 1). This suggests that the occurrence of *NF2* mutations in PM is independent of these demographic and clinical factors, pointing to other biological mechanisms driving these genetic alterations.

**Table 1:** Clinical and genetic features of PM patients with *NF2* mutations in the TCGA cohort

Histological subtype	Diagnosis age	Neoplasm stage	Protein change	Mutation type	Variant type	Allele Freq (T)	Variant reads	ClinVar/Varsome
Epithelioid	68	Stage III	X39_splice	Splice_Site	SNP	0.18	5	P/P
Epithelioid	75	Stage II	Y144*	Nonsense_Mutation	INS	0.27	12	P/P
Undefined	64	Stage II	R57*	Nonsense_Mutation	SNP	0.52	14	P/P
Biphasic	67	Stage III	E463*	Nonsense_Mutation	SNP	0.19	11	P/P
Biphasic	61	Stage IV	E465*	Nonsense_Mutation	SNP	0.32	34	NR/LP
Epithelioid	67	Stage III	Y192*	Nonsense_Mutation	SNP	0.26	6	NR/LP
Epithelioid	47	Stage II	X81_splice	Splice_Site	SNP	0.27	21	P/P
Biphasic	73	Stage III	X333_splice	Splice_Site	SNP	0.83	15	NR/P
Biphasic	57	Stage IV	W184*	Nonsense_Mutation	SNP	0.2	8	P/LP
Epithelioid	36	Stage IV	Q337*	Nonsense_Mutation	SNP	0.37	20	NR/LP
Epithelioid	46	Stage II	X121_splice	Splice_Site	SNP	0.1	3	NR/LP
Biphasic	63	Stage III	K413*	Nonsense_Mutation	SNP	0.24	15	NRLP
Epithelioid	66	Stage I	X482_splice	Splice_Site	SNP	0.5	19	NR/LP
Biphasic	58	Stage II	X447_splice	Splice_Site	SNP	0.71	10	P/LP
Epithelioid	55	Stage III	S87Gfs*15	Frame_Shift_Del	DEL	0.44	20	NR/LP
Epithelioid	54	Stage IV	R187Lfs*21	Frame_Shift_Del	DEL	0.22	8	NR/LP
Undefined	66	Stage IV	V24*	Frame_Shift_Del	DEL	0.13	35	NR/LP
Epithelioid	68	Stage III	K449Rfs*45	Frame_Shift_Del	DEL	0.24	13	P/P
Biphasic	68	Stage III	Y153*	Frame_Shift_Del	DEL	0.71	56	NR/LP
Epithelioid	62	Stage IV	G161Dfs*13	Frame_Shift_Del	DEL	0.14	7	NR/LP

*P* pathogenic, *LP* likely pathogenic, *NR* not reported in Clinvar

### Association of clinicopathological variables with NF2 mutational status  

We conducted a bivariate logistic regression analysis to assess the potential associations between various clinicopathological features of PM patients and the presence of *NF2* mutations. The analysis indicated that the evaluated clinicopathological features, including demographic factors, detection methods, primary diagnosis, tumor stage, and cancer status, showed no significant association with *NF2* mutational status in PM patients (Supplementary Table 1).

### Methylation, mutational and expression status of NF2 gene, and overall survival in the PM TCGA cohort

**Fig. 2:**
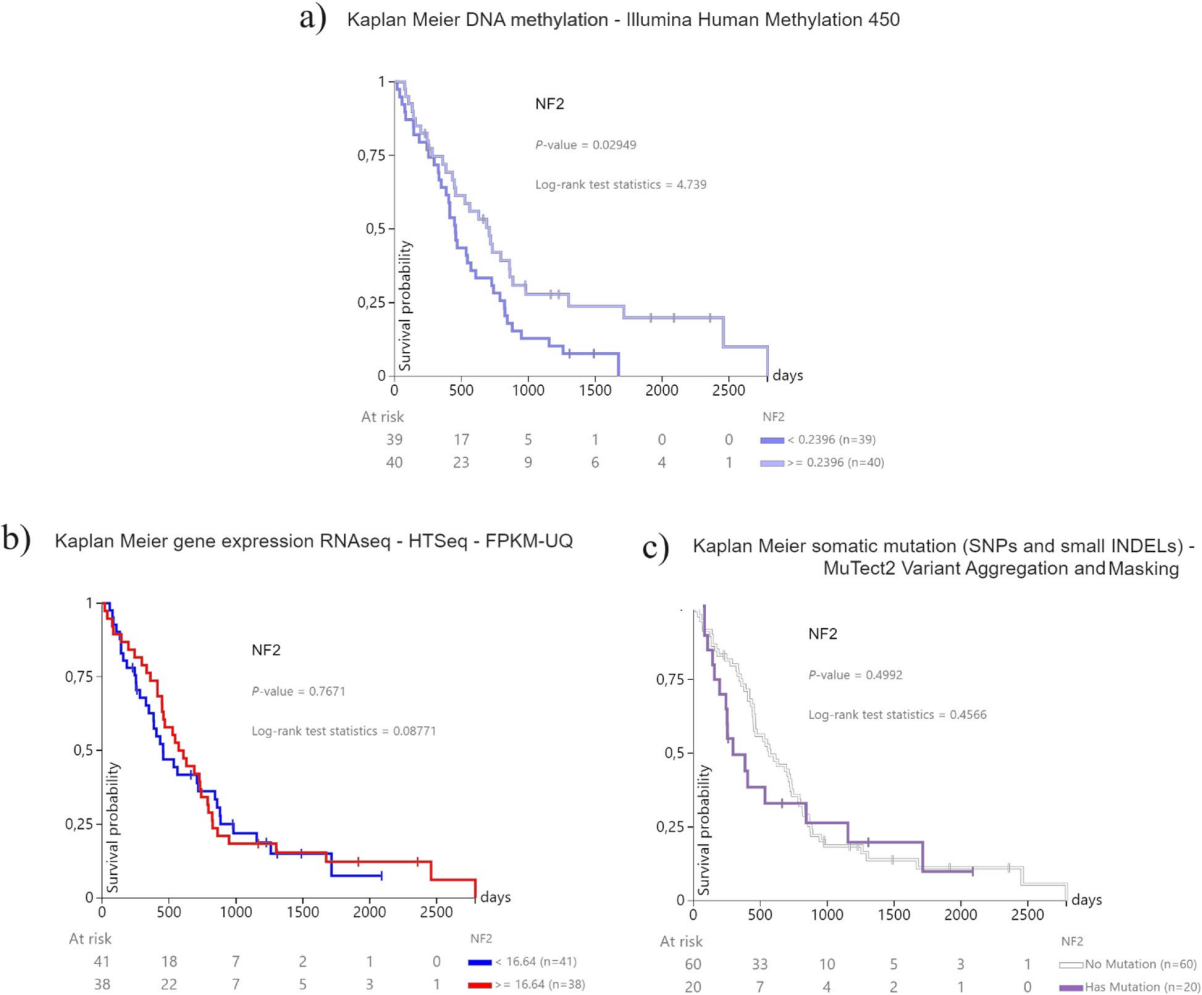
Association between *NF2* status and overall survival in PM patients: Kaplan–Meier survival analyses stratified by **a** NF2 methylation status (*p* = 0.0295), **b** NF2 expression levels (*p* = 0.7671), and **c** *NF2* mutational status (*p* = 0.4992). Plots show overall survival differences, with statistical significance considered at *p* < 0.05. Generated using the Xena Browser survival module

We analyzed the overall survival (OS) of patients with PM based on *NF2* methylation status using Kaplan–Meier survival curves (Fig. 2a). Patients were stratified into two groups according to their *NF2* methylation levels (≤ 0.2396 and > 0.2396). The results indicated a statistically significant difference in survival between the two groups, with a *p*-value of 0.02949 and a log-rank test statistic of 4.739. Patients with lower *NF2* methylation levels (≤ 0.2396) exhibited improved survival compared to those with higher methylation levels (> 0.2396). We then investigated the impact of *NF2* expression levels on OS (Fig. 2b). Patients were divided into two groups based on *NF2* expression levels (≤ 1.664 and > 1.664). The Kaplan–Meier analysis revealed no significant difference in survival between patients with low and high *NF2* expression levels, with a *p*-value of 0.7671 and a log-rank test statistic of 0.08771. This suggests that *NF2* expression levels do not significantly influence overall survival in this cohort. Finally, we assessed the association between *NF2* mutational status and OS (Fig. 2c). Patients were categorized based on the presence or absence of *NF2* mutations. The Kaplan–Meier survival curves demonstrated no significant difference in OS between the two groups, with a *p*-value of 0.4992 and a log-rank test statistic of 0.4566. These findings indicate that the presence of *NF2* mutations does not impact the overall survival of PM patients in this cohort.

### Differential expression analysis of PM patients regarding NF2 mutational status in the TCGA cohort

**Fig. 3:**
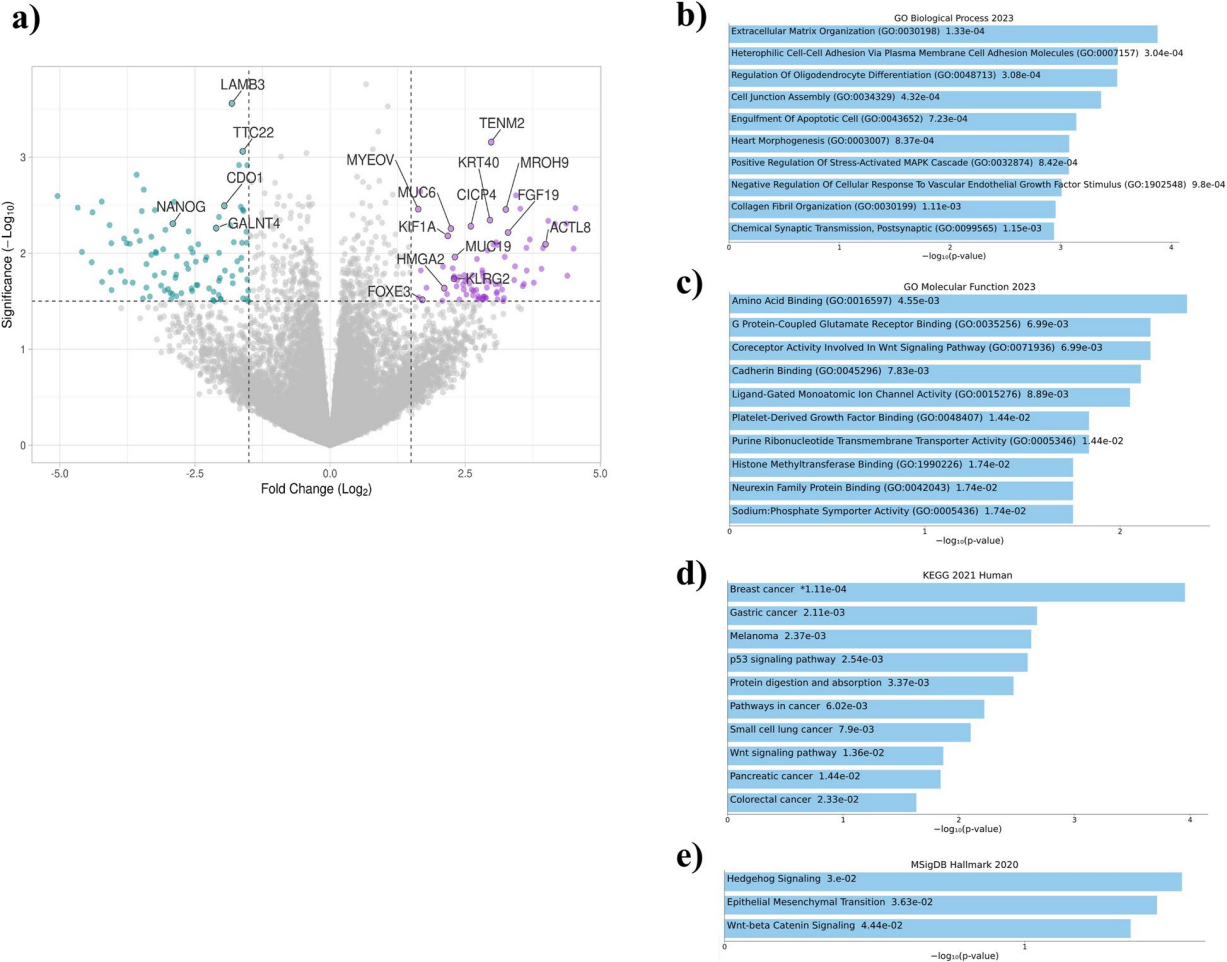
Differential expression analysis of PM TCGA patients. **a** Volcano plot illustrating differentially expressed genes in patients with *NF2* mutations. Upregulated genes are highlighted in violet, while downregulated genes are shown in blue. Enrichment analysis results for *NF2*-mutated patients, **b** GO biological processes, **c** GO molecular functions, **d** KEGG, and **e** MSigDB. This panel presents the results of the Gene Ontology enrichment analysis generated using Enrichr. The *x*-axis represents the -log10 (*p*-value) for each term. Only terms displaying significant differences are shown (*p* < 0.05)

We also investigated the transcriptional changes mediated by *NF2* mutations to gain a deeper understanding of their consequences on tumor biology. Although our survival analysis did not reveal a significant association between *NF2* mutational status or expression levels and OS, we observed that patients with hypomethylation of the *NF2* gene promoter had worse overall survival. This finding led us to further explore the transcriptional impact of *NF2* mutations in PM. To this end, we performed a differential expression analysis between *NF2-*mutated PM patients and those with wild-type *NF2* using data from the TCGA cohort. Our analysis identified a distinct set of genes that were significantly upregulated or downregulated in *NF2*-mutated patients compared to *NF2* wild-type patients (Fig. 3a). These transcriptional changes indicate potential pathways and processes altered by *NF2* mutations.

To better understand the biological features of *NF2* mutated patients, we conducted a functional enrichment analysis on the differentially expressed genes. This analysis revealed enrichment in several key pathways, including those involved in cell cycle regulation, apoptosis, and signal transduction. Notably, genes related to the Hippo signaling pathway, which is known to be regulated by NF2, were significantly affected, supporting the role of NF2 loss in the dysregulation of this pathway in PM. The insights gained from these analyses underscores the substantial biological alterations driven by *NF2* mutations and highlights the need for further research into targeted therapies addressing these specific changes. Several of the upregulated genes identified in the analysis (Fig. 3b–e) are involved in common cellular processes, including cell proliferation, differentiation, structural integrity, intracellular transport, extracellular matrix organization, and immune response regulation. The upregulation of these genes in *NF2-*mutated PM patients underscores the significant biological changes induced by NF2 loss, pointing to potential therapeutic targets. Transcription factors and kinases whose targets are overrepresented among the upregulated and downregulated genes are presented in SupplementaryTable 2.

### Survival analysis from top differentially expressed genes on NF2-mutated patient

**Fig. 4:**
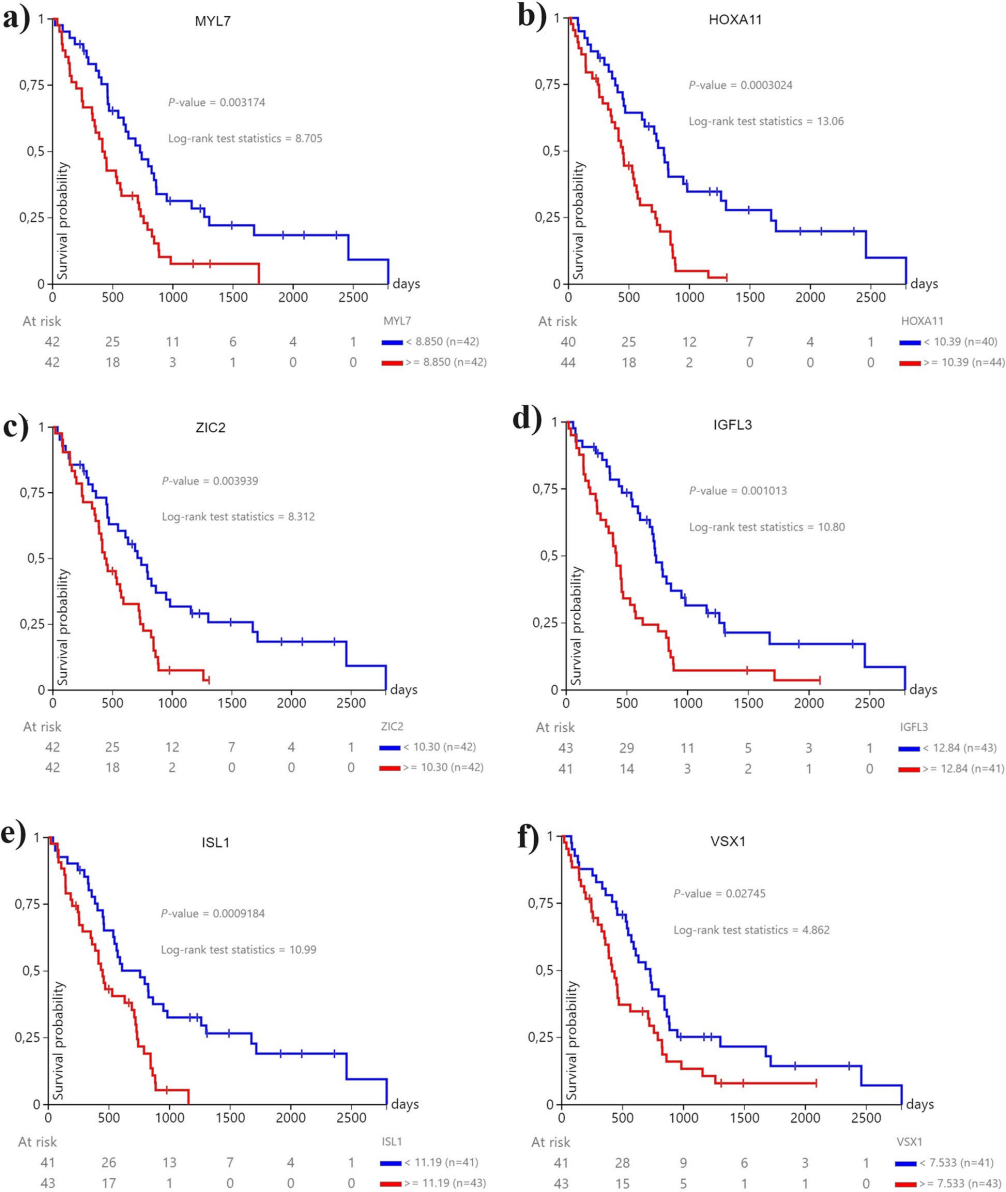
Kaplan–Meier survival curves for differentially expressed genes in TCGA PM patients with *NF2* gene mutations. This figure presents Kaplan–Meier survival curves for differentially expressed genes identified in PM patients harboring *NF2* gene mutations, using data from The TCGA. The survival curves correspond to genes that demonstrated significant associations in the Cox univariate proportional hazard model. The genes analyzed include **a** *MYL7*, **b** *HOXA11*, **c** *ZIC2*, **d** *IGFL3*, **e** *ISL1*, and **f** *VSX1* prognostic values were determined by a *p*-value threshold of < 0.05 in the log-rank test

To investigate the impact of upregulated DEGs on the survival of PM patients with *NF2* mutations, we performed a series of analyses using data from The TCGA. We compared the gene expression profiles of *NF2-*mutated PM patients with those of patients with wild-type *NF2* to identify potential prognostic markers. Initially, we conducted a univariate Cox regression analysis on the top 40 upregulated DEGs identified in the *NF2-*mutated group. Our analysis revealed that 13 DEGs were significantly associated with hazard ratios (HR > 1.2) and had *p*-values < 0.05, indicating that higher expression of these genes correlated with reduced OS (Supplementary Table 3). To further assess the prognostic value of these DEGs, we performed a multivariate Cox regression analysis. Of the 13 DEGs identified in the univariate analysis, five maintained statistical significance after adjusting for the expression levels of all 12 genes (Supplementary Table 4). In addition, to evaluate the prognostic potential of these DEGs, we stratified patients into high- and low-expression groups based on the median expression level of each gene. We then performed Kaplan–Meier survival analyses to determine whether these expression groups correlated with differences in survival outcomes. The results showed that higher expression levels of six genes were significantly associated with poorer survival outcomes, as indicated by log-rank test *p*-values < 0.05 (Fig. 4). Overall, our findings indicate that the upregulated expression *MYL7, HOXA11, ZIC2, IGFL3, ISL1,* and *VSX1* in *NF2-*mutated PM patients is associated with decreased overall survival, highlighting their potential role as prognostic biomarkers in this patient cohort.

### Estimation of the immune and stromal cell infiltration by deconvolution of transcriptional data from the PM TCGA cohort

**Fig. 5:**
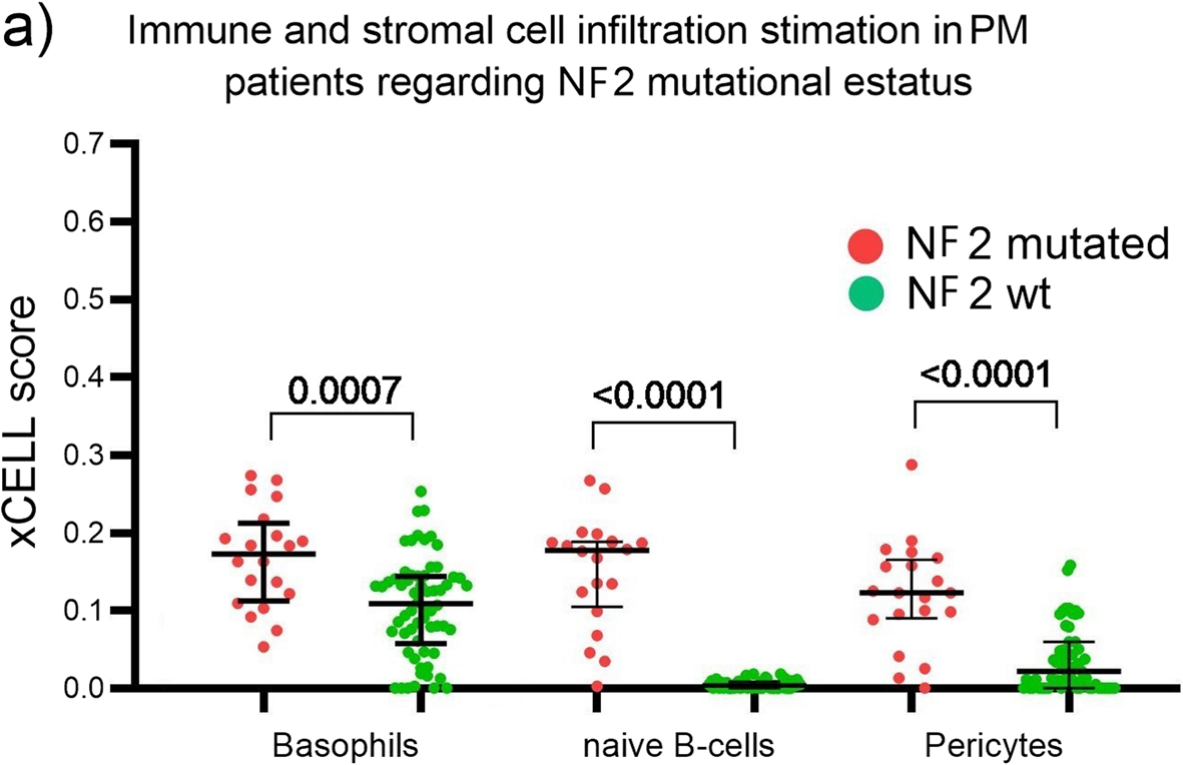
Results from the xCell deconvolution algorithm for estimation of immune and stromal cells in the PM TCGA cohort based on *NF2* mutational status. This figure presents the results of the xCell deconvolution analysis, estimating the presence of immune and stromal cells in the PM TCGA cohort with respect to *NF2* mutational status. Statistical significance was observed in 2 immune cells (basophils and naive B-cells) and 1 stromal cell (pericytes). The figure was generated using GraphPad. *P*-values were calculated using the Mann–Whitney *U* test, with significance considered at *p* < 0.05

We investigated whether *NF2* mutations were associated with differences in immune and stromal cell infiltration in PM tumors. To address this, we applied the xCell deconvolution algorithm to infer the infiltration of various cell types in *NF2*-mutated and wild-type tumors. Among the 64 cell types analyzed, significant differences were observed in the infiltration levels of basophils, naïve B cells, and pericytes. In *NF2-*mutated patients, the infiltration of all three cell types was significantly increased compared to their wild-type counterparts (Fig. 5).

### Prediction of chemotherapeutic response of PM patients from the TCGA cohort

**Fig. 6:**
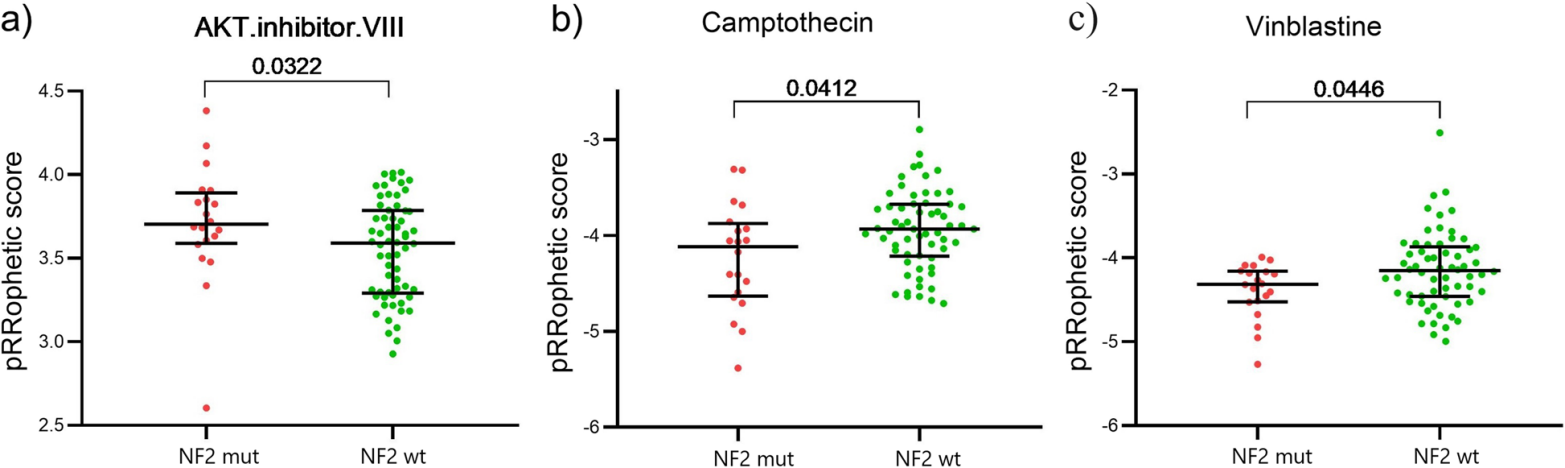
Prediction of clinical chemotherapeutic response from tumor gene expression levels in the PM TCGA cohort. **a** AKT Inhibitor VIII, **b** Camptothecin, **c** Vinblastine. *NS2*-mutated group (NF2 mut, *n* = 20) and *NS2* wild-type group (NF2 wt, *n* = 62). The analysis was conducted using the *pRRophetic* R package. The figure was generated using GraphPad. *P*-values were calculated using the Mann–Whitney *U* test, with significance considered at *p* < 0.05

To predict differences in chemotherapeutic response between *NF2-*mutated and *NF2* wild-type patients, we analyzed transcriptome data using the R package pRRophetic. Our analysis showed that *NF2-*mutated patients exhibited a modest but significant increase in sensitivity to camptothecin and vinblastine compared to *NF2* wild-type patients (*p* = 0.0412 and *p* = 0.0446, respectively) (Fig. 6). Conversely, *NF2* wild-type patients demonstrated higher sensitivity to Akt Inhibitor VIII compared to those with *NF2* mutations (*p* = 0.0322). These findings suggest that *NF2* wild-type tumors may respond more favorably to Akt inhibition therapy.

### Differential expression of cytokines, chemokines, and growth factors in NF2-mutated versus wild-type PM

**Fig. 7:**
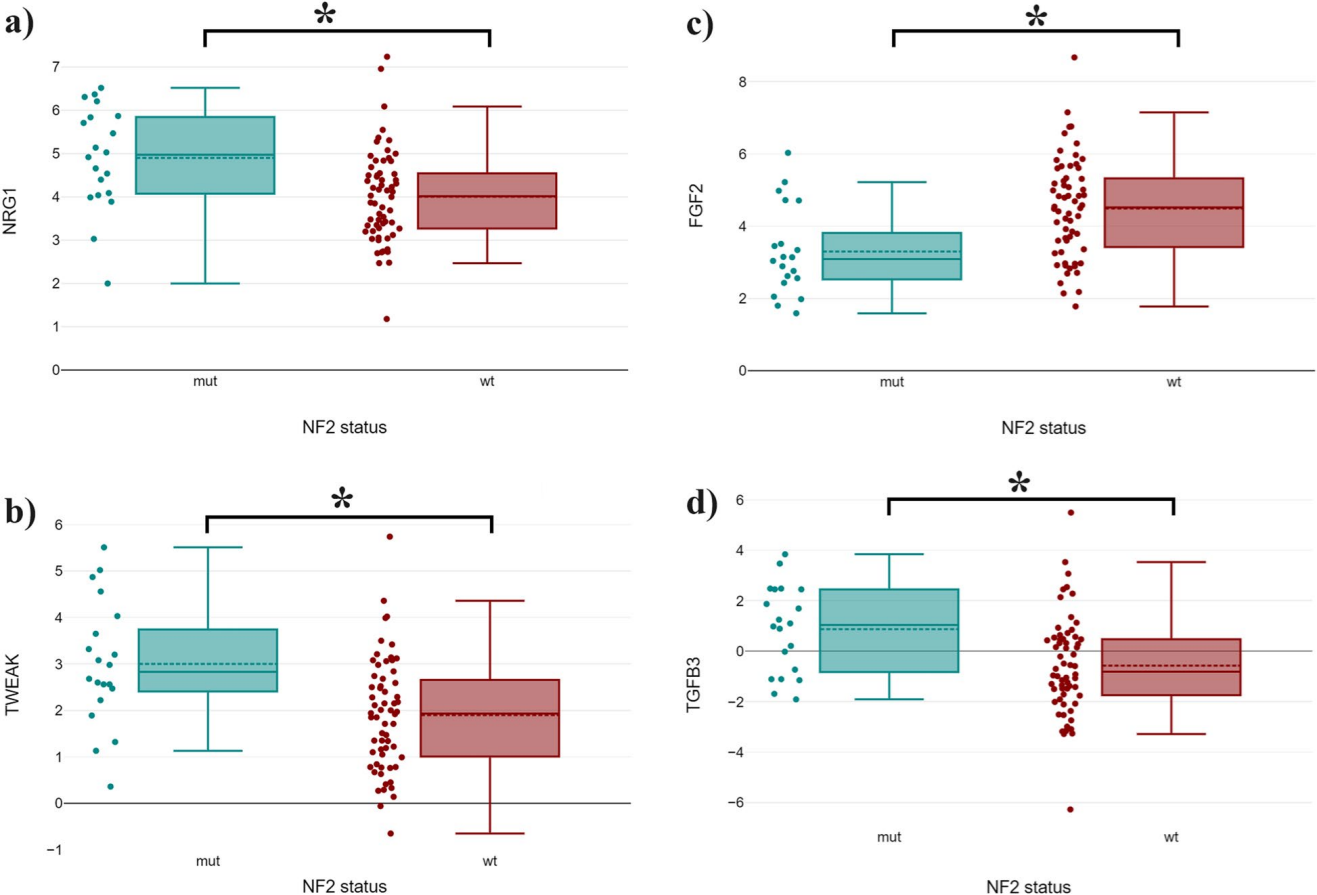
Differential expression of cytokines, chemokines, and growth factors regarding *NF2* status. Box plots illustrating the expression levels of four significantly altered molecules in *NF2*-mutated and *NF2* wild-type PM patients: **a** NRG1, elevated expression in *NF2-*mutated patients. **b** TWEAK, increased expression in *NF2*-mutated patients. **c** FGF2, reduced expression in *NF2-*mutated patients. **d** TGFB3, higher expression in *NF2-*mutated patients. Statistical significance was denoted by an asterisk (*p* < 0.05)

We investigated the potential changes in cytokines, chemokines, and growth factors between *NF2*-mutated and wild-type PM patients. To achieve this, we utilized the Cytosig deconvolution tool to identify variations between the two groups. Among the 64 cytokines, chemokines, and growth factors analyzed, we found significant differences in four molecules: NRG1, FGF2, TNF- TWEAK, and TGFB3 (Fig. 7). Our results indicate that *NF2-*mutated patients have higher levels of NRG1, TWEAK, and TGFB3, but lower levels of FGF2 compared to *NF2* wild-type patients.

### Single cell analysis

**Fig. 8:**
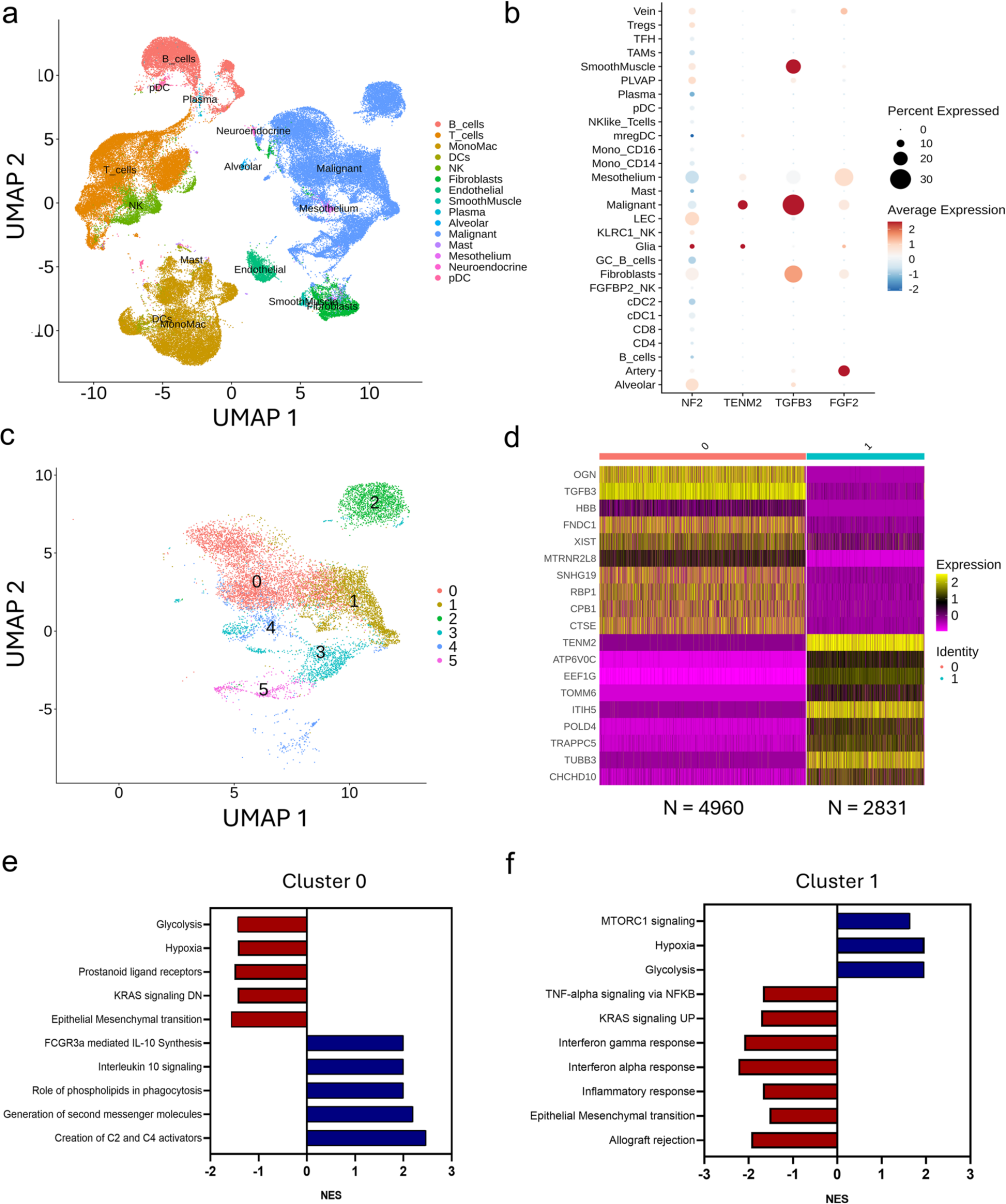
Single-cell RNA sequencing analysis of PM samples. **a** UMAP projection of cells colored by cell type, showing the distribution and clustering of different cell types in the tumor microenvironment. **b** Dot plot of gene expression for *NF2*, *TENM2*, *TGFB3*, and *FGF2* across different cell types, illustrating the variability in gene expression profiles. **c** UMAP projection colored by cluster number, indicating subpopulations of malignant cells expressing TENM2, TGFB3, and FGF2 and no expression of NF2. **d** Heatmap of differentially expressed genes across clusters, highlighting Cluster 0 with overexpression of TGFB3 and Cluster 1 with high levels of TENM2. **e** Pathway enrichment analysis for Cluster 0, showing upregulated (blue) and downregulated (red) pathways with normalized enrichment scores (NES). **f** Pathway enrichment analysis for Cluster 1, indicating significant biological processes and signaling pathways active in these cells

We performed a single-cell RNA sequencing analysis on data from PM samples, including 13 primary tumors and 84,526 cells [25]. This analysis revealed distinct cellular subpopulations and their gene expression profiles (Fig. 8). The UMAP projection (Fig. 8a) showed a diverse array of cell types within the tumor microenvironment, including B cells, T cells, NK cells, monocytes/macrophages, dendritic cells, fibroblasts, and various mesothelial and malignant cell populations. We further investigated the expression patterns of NF2, TENM2, TGFB3, and FGF2 across these cell types. As shown in Fig. 8b, the dot plot indicates that *NF2* expression is notably higher in malignant and mesothelial cells compared to other cell types. In contrast, genes such as TGFB3 and FGF2 were expressed across multiple cell types, suggesting their involvement in various cellular processes within the tumor microenvironment.

To explore the heterogeneity of malignant cells, we clustered those that do not express *NF2* but do express at least one of the following genes: TENM2, TGFB3, and FGF2 (Fig. 8c). This analysis identified five distinct clusters. Cluster 0 displayed high expression of TGFB3, which is also overexpressed in TCGA samples with *NF2* mutations (Fig. 8d). Cluster 1 showed elevated levels of TENM2, which is the most significantly overexpressed gene in TCGA samples with *NF2* mutations (Fig. 8a and d). Moreover, we identified other DEGs across these clusters. Notable genes include *OGN*, *CPB1*, and *XIST* in Cluster 0, and *ITH5*, *TUBB3*, and *EEF1G* in Cluster 1. Pathway enrichment analysis provided further insights into the functional differences between these clusters. Cluster 0 is characterized by IL-10 synthesis, production of C2 and C4 activators, and second messenger molecules (Fig. 8e), suggesting immune regulation by a subset of malignant cells. In contrast, Cluster 1 exhibited significant upregulation of mTORC1 signaling, hypoxia, and glycolysis (Fig. 8f), indicating metabolic reprogramming that may contribute to cell survival in the hypoxic tumor microenvironment.

Moreover, we identified intratumor heterogeneity of NF2 expression within patients (Supplementary Fig. 1). Patients displayed expressions of NF2 ranging from 0 to 16% of their malignant cells. We visually investigated the expression of NF2 in every sample and found that these tumor cells are spread along the malignant cells cluster, with no evidence of sub-clustering.

Finally, our sub-clustering analysis identified four different groups of B cells (SupplementaryFig. 2). Cluster 0 overexpressed IgM, consistent with activated B cells. Cluster 1 co-expressed IgM and IgD, and enrichment analysis demonstrated a lack of antigen binding, consistent with naive B cells. This cluster exhibited pathways related to angiogenesis and epithelial-to-mesenchymal transition. Cluster 2 was enriched for proliferative markers, indicating cells undergoing mitosis, which could be an intermediate state between clusters 1 and 0.

## Discussion

In this study, we investigated the prevalence and impact of *NF2* mutations on various clinicopathological features, immune and stromal cell infiltration, and chemotherapeutic response in PM patients using available bulk and single-cell RNA seq data. Our key findings include a significant subset of PM patients harboring *NF2* mutations, distinct transcriptional landscapes associated with these mutations, and differential chemotherapeutic responses based on *NF2* status. We observed that *NF2* mutations were independent of traditional clinicopathological factors such as histological subtype, tumor stage, and age. Notably, *NF2-*mutated tumors exhibited increased infiltration of basophils, naïve B cells, and pericytes. Furthermore, predictive modeling indicated that *NF2-*mutated patients showed increased sensitivity to camptothecin and vinblastine, while *NF2* wild-type patients were more responsive to Akt Inhibitor VIII. This study supports previous research highlighting the significance of genetic alterations in PM and offers new insights that could guide future preclinical therapeutic developments [8, 9, 26].

Our analysis of the TCGA PM cohort revealed that *NF2* mutations are present in 20 out of 82 patients (Fig. 1), underscoring the gene’s frequent alteration in PM. These mutations primarily result in loss of function, impairing Merlin’s ability to regulate cell proliferation and maintain cellular homeostasis. This disruption of the Hippo pathway facilitates uncontrolled cell growth and contributes to tumorigenesis in PM [5, 12, 27, 28]. The high frequency of *NF2* mutations observed is consistent with prior research, emphasizing the gene’s critical role in PM pathogenesis. For example, Bueno et al. [3] reported similar findings, with *NF2* mutations identified in 22% of PM cases, reinforcing the gene’s importance in the disease. Our findings also align with those of Quetel et al. [8] who highlighted the association between *NF2* mutations and distinct molecular subtypes of PM. This suggests that these genetic alterations contribute to tumor heterogeneity and influence disease progression. Although *NF2* mutational status and gene expression levels were not associated with overall survival in the TCGA PM cohort, hypomethylation of the *NF2* promoter correlated with improved survival (Fig. 2), reinforcing the relevance of epigenetic regulation in its function. DNA methylation is a key transcriptional regulator, but its effects extend beyond direct gene silencing. Hypomethylation may enhance *NF2* activity through mechanisms such as increased chromatin accessibility or interaction with regulatory elements that influence tumor suppressor pathways. These findings suggest that epigenetic modifications, rather than NF2 mRNA levels alone, may play a critical role in PM progression and patient outcomes. Further studies are needed to clarify the functional consequences of *NF2* methylation in this context.

The impact of *NF2* mutations extends beyond their role as tumor suppressors. Studies have shown that *NF2* mutations can affect the tumor microenvironment by influencing immune cell infiltration and stromal interactions. These effects have significant implications for both diagnostics and prognostics. For example, Johnson and Halder [7] highlighted that *NF2* mutations lead to dysregulation of the Hippo pathway, which is involved in cell proliferation, apoptosis, and stem cell renewal. Our findings, which reveal increased immune and stromal cell infiltration in *NF2* mutated tumors, support this view and suggest that *NF2* mutations may create a tumor microenvironment that favors tumor growth and immune evasion.

*NF2* mutations in PM patients were not significantly associated with histological subtype, tumor stage, or age (Supplementary Table 1), suggesting that these alterations occur independently of traditional clinical features. This finding aligns with previous reports [8, 9], though other studies have noted higher *NF2* mutation rates in biphasic and sarcomatoid tumors, implicating a role in epithelial–mesenchymal transition [29]. The limited number of non-epithelioid cases in the TCGA MESO cohort may have reduced our ability to detect such associations. Further studies with more balanced histological representation are needed to clarify this relationship.

Differential expression analysis revealed a distinct transcriptional landscape in *NF2* mutant PM tumors, with enrichment in pathways related to extracellular matrix organization, cell adhesion, and apoptosis (Fig. 3b). These alterations may underline the aggressive phenotype of *NF2* mutant tumors. Supporting our findings, Xu et al. [30] showed that NF2/LATS1/2-deficient PM cells are more sensitive to the BCL-XL inhibitor A-1155463, suggesting a therapeutic vulnerability via modulation of apoptotic pathways, particularly through BH3 mimetics. Additionally, pathways associated with cancer, such as the p53 signaling pathway and those specific to breast and gastric cancer, were significantly enriched in *NF2-*mutated cases (Fig. 3d). This highlights potential oncogenic processes that may be shared across different tumor types. Moreover, hallmark pathways like epithelial-mesenchymal transition and Wnt-beta catenin signaling (Fig. 3e) were implicated, aligning with previously reported mechanisms where *NF2* loss enhances tumor invasiveness and resistance to cell death [31, 32]. These findings provide a deeper understanding of the molecular alterations in *NF2-*mutated PM and could inform future therapeutic strategies targeting these pathways.

The deconvolution analysis of cytokines and growth factors indicates that *NF2* mutations are linked to specific transcriptional programs, which may contribute to the pathogenesis and progression of PM (Fig. 7). Key upregulated cytokines and growth factors in *NF2-*mutated tumors include NRG1, TWEAK, and TGFB3. NRG1 is known to play a role in cell growth, differentiation, and survival through activation of the ERBB receptor family, suggesting a potential mechanism by which *NF2* mutations promote tumor growth and resistance to apoptosis [33]. TWEAK, a member of the TNF superfamily, has been implicated in tumor cell proliferation, migration, and angiogenesis, which supports its role in the aggressive behavior of *NF2-*mutated PM [34]. TGFB3 is involved in regulating cell growth and differentiation, and its upregulation in *NF2-*mutated tumors may contribute to the modulation of the tumor microenvironment and immune evasion [35].

The transcriptional changes induced by *NF2* mutations have substantial implications for the biology of PM and the development of potential therapeutic targets (Fig. 3). The upregulation of genes associated with growth factor signaling and immune modulation suggests that targeting these pathways may offer a viable therapeutic strategy. For instance, inhibitors of the ERBB receptor family, which are activated by NRG1, could be explored as potential treatments for *NF2-*mutated PM. Additionally, targeting the Hippo pathway and its downstream effectors represents another promising therapeutic approach. Drugs that restore the function of the Hippo pathway or inhibit the overactive cell cycle and proliferation signals may prove effective in treating *NF2-*mutated PM [36].

Our study employed the xCell deconvolution algorithm to estimate the relative abundance of various immune and stromal cell types in *NF2*-mutated and wild-type PM tumors (Fig. 5). The analysis revealed significant differences in the infiltration levels of basophils, naïve B cells, and pericytes. Specifically, *NF2-*mutated tumors exhibited increased infiltration of these cell types compared to *NF2* wild-type tumors, suggesting that *NF2* mutations may influence the composition of the tumor microenvironment. Basophils, which are white blood cells involved in inflammatory responses and the release of histamine, were found in greater numbers in *NF2-*mutated tumors. This increased presence suggests a potential role in modulating the tumor immune microenvironment. Basophils may influence tumor progression and metastasis by promoting an immunosuppressive environment that facilitates tumor cell evasion from immune detection. Additionally, basophils can release factors that support angiogenesis and tissue remodeling, which are crucial for tumor growth [37]. Naïve B cells, which are immature B lymphocytes not yet exposed to antigens, were also significantly elevated in *NF2-*mutated tumors. In the tumor microenvironment, naïve B cells can differentiate into various subsets that either support anti-tumor immune responses or contribute to an immunosuppressive niche that protects the tumor from immune attacks. This dual role of B cells underscores the complexity of their involvement in cancer biology [38]. The observed increase in naïve B cells in mutated tumors may be linked to dysregulation of the Hippo signaling pathway caused by NF2 loss. Inactivation of NF2 leads to aberrant activation of YAP/TAZ, transcriptional co-activators that modulate cytokine and chemokine expression, potentially influencing the recruitment and retention of immune cell subsets, including B cells [39]

Our predictive modeling of chemotherapeutic response, using the pRRophetic R package, revealed significant but modest differences in drug sensitivity between *NF2*-mutated and wild-type PM patients. Specifically, *NF2*-mutated patients demonstrated increased sensitivity to camptothecin and vinblastine, whereas *NF2* wild-type patients exhibited higher sensitivity to Akt Inhibitor VIII (Fig. 6). These findings should be further investigated in preclinical studies to emphasize the importance of *NF2* mutation status in guiding personalized therapy in PM. Camptothecin, a topoisomerase I inhibitor, and vinblastine, a microtubule inhibitor, both show increased efficacy in *NF2*-mutated PM patients. Camptothecin induces DNA damage by stabilizing the topoisomerase I-DNA complex, leading to DNA breaks during replication. The heightened sensitivity of *NF2-*mutated tumors to camptothecin suggests that these tumors may have compromised DNA repair mechanisms, making them more susceptible to DNA-damaging agents [40]. This aligns with previous studies indicating that disruptions in the *NF2* gene can impair the cell’s ability to repair DNA damage, thereby enhancing the cytotoxic effects of camptothecin [36]

Conversely, *NF2* wild-type patients exhibited higher sensitivity to Akt Inhibitor VIII, a selective inhibitor of the Akt signaling pathway. The PI3K/Akt/mTORC1 pathway is a crucial regulator of cell growth, survival, and metabolism, with its dysregulation frequently observed is various cancers. In *NF2* wild-type tumors, the Akt pathway may play a more significant role in driving tumor growth and survival. Therefore, targeting this pathway with Akt inhibitors could be particularly effective for these patients. This differential sensitivity highlights the potential for incorporating Akt inhibitors into a personalized therapy approach for *NF2* wild-type PM patients [41].

Single-cell analysis identified a cluster of malignant cells characterized by high *TENM2* expression and activation of mTORC1, glycolysis, and hypoxia pathways (Fig. 8f). These pathways are upregulated in *NF2-*mutated cells [39, 42]. mTORC1 regulates global protein synthesis in response to nutrients and growth factors, promoting cell growth and division [43]. Cancer cells often display altered metabolism, including increased glucose uptake and the fermentation of glucose to lactate (Warburg effect) [44]. Glycolysis produces ATP more rapidly than oxidative phosphorylation and contributes to tumor growth, invasion, and chemoresistance [45]. Moreover, hypoxia reprograms tumor cells to increase glycolysis, demonstrating the interconnection between these pathways [46]. Collectively, cluster 1 exhibits phenotypic features consistent with *NF2-*mutated cells.

While our study provides valuable insights into the role of *NF2* mutations in PM, several limitations should be acknowledged. First, the analysis was based on publicly available datasets, which may not fully represent the genetic diversity of PM across different populations. Additionally, the relatively small sample size of the TCGA cohort, particularly the subset of patients with *NF2* mutations, limits the generalizability of our findings. The cross-sectional nature of the data also restricts our ability to draw causal inferences about the relationship between *NF2* mutations and clinical outcomes. Furthermore, although we utilized computational methods to estimate immune and stromal cell infiltration, these findings should be validated through experimental approaches in future studies. Lastly, while the prediction of chemotherapeutic responses based on gene expression profiles is suggestive, it requires clinical validation to confirm its potential for guiding personalized therapy in PM patients. Future research with larger cohorts, longitudinal designs, and experimental validation is necessary to address these limitations and reinforce the findings of our study.

In conclusion, this study enhances our understanding of the molecular mechanisms underlying PM and underscores the significance of *NF2* mutations as biomarkers for disease progression and therapeutic response. The findings offer a basis for further research and the development of more effective, personalized therapies, ultimately aiming to enhance the prognosis and quality of life for PM patients.

## Supplementary Information

Supplementary Material 1.

Supplementary Material 2.

Supplementary Material 3.

Supplementary Material 4.

Supplementary Material 5.

Supplementary Material 6.

## Data Availability

The datasets analyzed during the current study are publicly available. Molecular data from the 82 MPM patients were accessed through the UCSC Xena Browser and can be retrieved from https://xenabrowser.net/datapages/?cohort=GDC%20TCGA%20Mesothelioma%20(MESO)&removeHub=https%3A%2F%2Fxena.treehouse.gi.ucsc.edu%3A443. Single-cell RNA sequencing data were obtained from the Gene Expression Omnibus (GEO) under accession number GSE190597, accessible at https://www.ncbi.nlm.nih.gov/geo/query/acc.cgi?acc=GSE190597. These datasets provide the data necessary to replicate and interpret the findings of this study.

## Abbreviations

PM: Pleural mesothelioma
TCGA: The Cancer Genome Atlas
ACMG: Classified according to the American College of Medical Genetics
RNA-seq: RNA sequencing
GEO: Gene Expression Omnibus
UMI: Unique molecular identifier
PCA: Principal component analysis
UMAP: Uniform Manifold Approximation and Projection
DEGs: Differentially expressed genes
GO: Gene Ontology
BP: Biological process
MF: Molecular function
KEGG: Kyoto Encyclopedia of Genes and Genomes
NES: Normalized Enrichment Scores
OS: Overall survival
HR: Hazard ratio
