# Correction: Transcriptional landscape of pleural mesothelioma patients in relation to *NF2* gene mutational status

**DOI:** 10.1186/s43046-025-00310-1

**Published:** 2025-07-03

**Authors:** Carlos Orozco‑Castano, Alejandro Mejia‑Garcia, Hsuan Megan Tsao, Diego A. Bonilla, Carlos Carvajal-Fierro, Ricardo Bruges‑Maya, Alba Combita, Rafael Parra‑Medina

**Affiliations:** 1 Instituto Nacional de Cancerologia, Bogota, Colombia; 2 McGill University, Montreal, Canada; 3 University of the Basque Country, Leioa, Spain; 4 DBSS International SAS, Bogota, Colombia; 5 Fundacion Universitaria de Ciencias de La Salud, Bogota, Colombia

**Correction** **:** **J Egypt Natl Canc Inst 37, 25 (2025)**

**https://doi.org/10.1186/s43046-025-00284-0**

Following publication of the original article [1], the author reported that the 5th author was omitted from the author group. **Carlos Carvajal-Fierro** has been added to the author group and are presented correctly in this correction article.

The original article [1] has been corrected.
