# Supplementary figures and images for: Transcriptional landscape of pleural mesothelioma patients in relation to NF2 gene mutational status
Source: J Egypt Natl Canc Inst. 2025 Jun 9;37:25. doi: 10.1186/s43046-025-00284-0 (PMC13313441; doi:10.1186/s43046-025-00284-0)

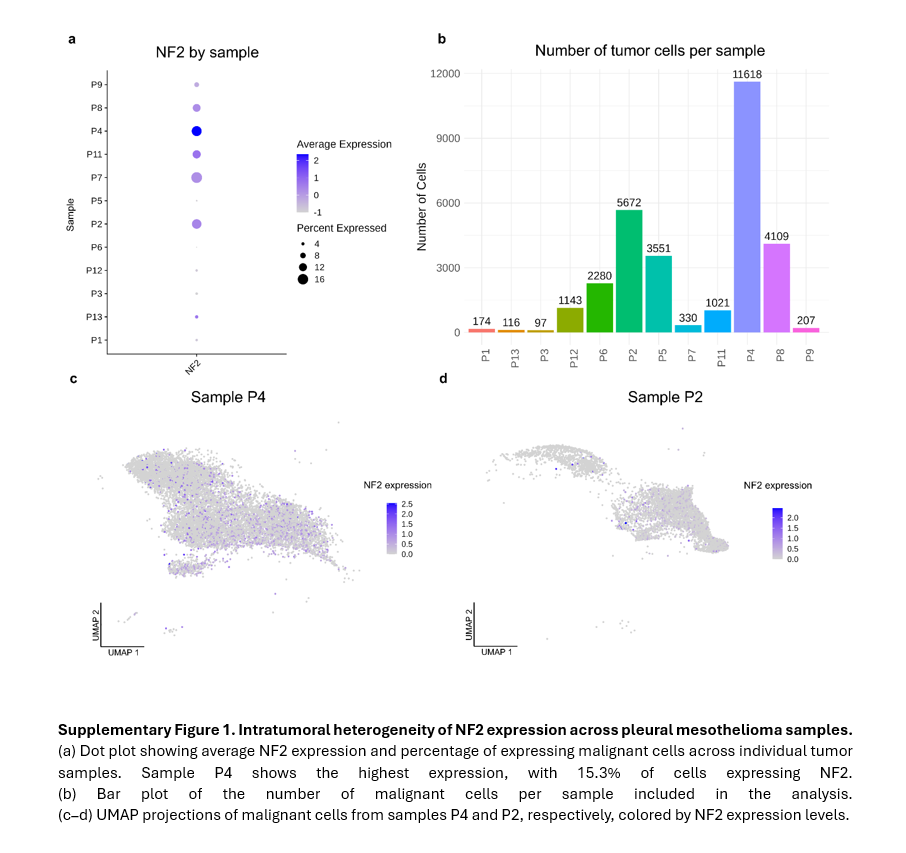

Supplement: Supplementary file 5 — Supplementary Material 5. [file 43046_2025_284_MOESM5_ESM.png]

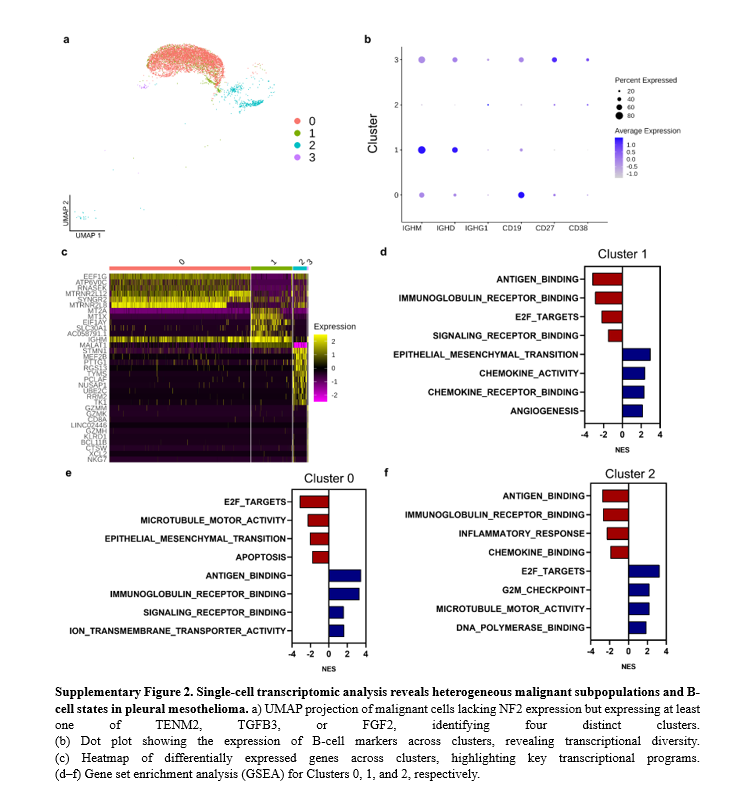

Supplement: Supplementary file 6 — Supplementary Material 6. [file 43046_2025_284_MOESM6_ESM.png]
